# Obesity is associated with suppressed bone turnover: a systematic review and meta-analysis

**DOI:** 10.3389/fphys.2026.1793838

**Published:** 2026-06-10

**Authors:** Haiqi Lin, Xiaohua Liu, Binglin Chen, Chengyi Liu

**Affiliations:** 1 School of Physical Education and Sports Science, South China Normal University, Guangzhou, Guangdong, China; 2 College of Physical Education, South China University of Technology, Guangzhou, Guangdong, China; 3 School of Exercise and Health, Shanghai University of Sport, Shanghai, China; 4 The Second School of Clinical Medical College, Xuzhou Medical University, Xuzhou, Jiangsu, China

**Keywords:** bone, bone formation, bone resorption, bone turnover (markers), obesity

## Abstract

**Background:**

Obesity is an increasingly serious public health issue on a global scale. Although there is increasing research on the relationship between obesity and biochemical markers of bone turnover, this relationship is complex and unclear. We conducted a systematic review and meta-analysis aimed at comparing the concentrations of biochemical markers of bone turnover between individuals with obesity and those with normal-weight.

**Method:**

A systematic search was conducted using the PubMed, EMBASE, Web of Science, Cochrane Library, and EBSCO databases from database inception to December 15, 2025. Two researchers screened the literature according to the inclusion and exclusion criteria and extracted valid data for quality evaluation.

**Results:**

The results ultimately included 14 articles with a total of 4612 subjects. Compared with normal-weight individuals, individuals with obesity showed no significant difference in alkaline phosphatase (ALP) levels. Procollagen type I N-terminal propeptide (PINP) levels were significantly reduced in individuals with obesity. Osteocalcin (OCN) levels were also decreased overall, but subgroup analysis showed that this reduction was significant in children and adolescents, whereas no significant difference was observed in adults. No significant difference was observed in C-terminal telopeptide of type I collagen (CTX-I) or N-terminal telopeptide of type I collagen (NTX) levels.

**Conclusion:**

This meta-analysis suggests that obesity is associated with suppressed bone turnover, mainly reflected by reduced levels of bone formation markers. PINP levels were significantly decreased in individuals with obesity, whereas the reduction in OCN appeared to be age-dependent, with significant suppression observed in children and adolescents but not in adults. However, the robustness of the bone resorption results is insufficient and requires further validation through additional studies. Further studies are needed to clarify the mechanisms by which obesity affects skeletal health and to identify potential targets for clinical intervention.

## 1 Introduction

Obesity is a chronic disease characterized by abnormal or excessive fat accumulation, and it poses a significant threat to human health. Obesity has become a serious public health problem worldwide, and it has gradually developed into an epidemic. More than 4 million people die from overweight or obesity every year (Bray et al., 2017; Burki, 2021). Studies have shown that obesity occurs at all stages of life, and its prevalence increases with age. More than half of middle-aged people in many countries are overweight or obesity, but experts are not optimistic about the prospect of reducing the prevalence of obesity (NCD Risk Factor Collaboration (NCD-RisC), 2016; The Lancet Public H, 2023). Obesity is the main determinant of disability and death, mainly because it increases the risk of cardiovascular disease, type 2 diabetes, osteoporosis, fatty liver, hypertension, myocardial infarction, osteoarthritis, cancer and other related diseases. It leads to a decrease in quality of life and life expectancy (Ceschi et al., 2007; Blüher, 2019; Tutor et al., 2023). Obesity not only affects the health of patients but also affects social sustainability by increasing health care costs and reducing labor market participation rates (Hjorth et al., 2023).

Bones constitute the framework of the body, provide the basic shape of the human body, and are important for protecting internal organs. During life, bone is constantly formed, absorbed and remodeled. Osteoclasts secrete proteinases to digest the bone matrix and secrete acidic substances to dissolve minerals, and osteoblasts secrete and mineralize bone matrix. The two are coupled and restrict each other, and they are in dynamic balance to cause bone turnover (Liu et al., 2023). Adipose tissue and bone are both metabolically active organs that continuously produce and release hormones and cytokines. The effects of adiposity on bone are mediated by a combination of mechanical and biochemical factors (Savvidis et al., 2018; Liu et al., 2023). Most researchers have explored different aspects of the complex link between obesity and bone conversion (Halade et al., 2011; Shapses et al., 2017; Ali et al., 2022). Previous studies have suggested that obesity is a protective factor for bone mass, which generates a strong gravitational load and mechanical stimulation, and activating the Wnt/β-catenin signaling pathway promotes bone formation and increases the bone density of load-bearing bones. Although most studies have shown a beneficial effect of obesity on bone density, this viewpoint has been continuously challenged in recent years (Mendonça et al., 2022). An increasing number of studies have shown that the positive effects of mechanical loading on bone health cannot offset the negative effects of hormones and inflammation. Excessive secretion of leptin and insufficient adiponectin by adipocytes in individuals with obesity may directly affect the bone formation signaling pathway or indirectly affect bone resorption by upregulating proinflammatory cytokines (Dib et al., 2014; Li et al., 2017). An increase in adipocytes in the bone marrow microenvironment can enhance the secretion of the inflammatory factors interleukin-6(IL-6) and tumor necrosis factor-α(TNF-α), which promotes osteoclast absorption by affecting the osteoprotegerin/receptor activator of nuclear factor-κB/receptor activator of nuclear factor-κB ligand (OPG/RANK/RANKL) signaling pathway (Wang and He, 2018). At the same time, adipocytes and osteoblasts originate from common bone mesenchymal stem cells (BMSCs). BMSCs can differentiate into both bone and fat. There is a trade-off between the two. That is, BMSCs differentiate more into adipocytes, which weakens their ability to differentiate into osteoblasts; in contrast, the differentiation of BMSCs into osteoblasts is enhanced, which weakens their ability to differentiate into adipocytes (Gimble and Nuttall, 2012). Studies have shown that obesity promotes adipocyte differentiation and thus fat accumulation and attenuates osteoblast differentiation (Proietto, 2020). Therefore, the relationship between obesity and bone mass is unknown.

Bone turnover biochemical markers are components of the bone matrix that are released into the blood and urine by the activity of osteoblasts and osteoclasts (Coates, 2013). In recent years, with increasing research on osteoporosis and metabolic bone diseases, biochemical markers of bone turnover have been found to play important roles in the diagnosis of a variety of bone diseases and the prediction of fracture risk (Eastell and Szulc, 2017; Tan et al., 2020). Jung et al. (2016) conducted differential analysis on the gene expression profiles of metabolic biomarkers and peripheral blood mononuclear cells in normal weight, mildly obesity, and moderately obesity subjects. The results showed that some genes may be linked to obesity-related metabolic disorders and bone metabolism disorders. However, the relationship between obesity and bone turnover is complex and unclear (Yerlikaya and Eryavuz Onmaz, 2022). The currently recognized biomarkers for bone metabolism are ALP, PINP, OCN, CTX-I, NTX, and deoxypyridinoline (DPD) (Coates, 2013; Eastell and Szulc, 2017; Brown et al., 2022). Serum PINP and CTX-I are the reference markers of bone formation and bone resorption identified by the International Osteoporosis Foundation (IOF) and are used to predict and monitor the risk of fracture therapy and osteoporosis treatment (Ferrante, 2007). Although ALP, OCN, DPD, and NTX have not been recommended as markers of bone formation and resorption, because the biochemical markers of bone turnover all have their own specific characteristics, their measurement together with one or more indicators and their combined analysis can provide a better basis for assessing changes in bone mineral density. The clinical detection of bone turnover biomarkers is highly sensitive, easy to obtain, repeatable, timely, and noninvasive; has a lower detection cost; and can reflect the treatment effect earlier than the current gold standard measurement of bone density. Bone turnover markers can be used for the differential diagnosis of osteoporosis, effectively predicting bone loss and fracture risk (Szulc, 2018; Schini et al., 2023). Obesity is a risk factor for many chronic diseases. The relationship between obesity and bone metabolism has been studied, but the results are diverse due to the differences among the studies. Therefore, this study aimed to explore the effect of obesity on the concentration of biochemical markers of bone turnover to investigate changes in bone homeostasis in individuals with obesity. This study has important guiding significance for improving the bone health status of individuals with obesity, guiding new prevention and treatment methods for obesity-induced osteoporosis, and preventing the occurrence of bone metabolism diseases.

This research program is registered on the PROSPERO platform (https://www.crd.york.ac.uk/PROSPERO/). The systematic review registration number is CRD42022347139.

## 2 Materials and methods

### 2.1 Data sources and search strategy

This protocol is registered with PROSPERO (CRD42022347139). The search was conducted using the PubMed, EMBASE, Web of Science, Cochrane Library, and EBSCO databases from the establishment of the databases to December 15, 2025. The search included a combination of MeSH terms and free text to search for clinical research articles related to obesity and bone blood biochemical indicators and was limited to English only. Taking the Web of Science database as an example, TS=(“obesity” OR “Body Mass Index” OR “fatty” OR “excess fat” OR “body fat*”) AND (“Osteogenesis” OR “Ossification*” OR “bone formation” OR “bone metabolism” OR “bone turnover” OR “Alkaline Phosphatase” OR “procollagen type I N-terminal peptide” OR “Osteocalcin” OR “bone resorption*” OR “osteoclastogenesis” OR “collagen type I trimeric cross-linked peptide” OR “deoxypyridinoline” OR “N-terminal telopeptide of type I collagen”) were selected (see **Supplementary File 1** for specific retrieval strategies).

#### 2.2.1 Inclusion criteria

PICOS methods (population, exposure, control, outcome, and study design) were used to define eligibility criteria (Liberati et al., 2009). The participants were individuals with obesity of any race, background, and age group, excluding individuals with overweight, postmenopausal women with obesity, and individuals with morbid obesity. No intervention was used to evaluate the relationship between obesity and biochemical indicators of bone formation and bone resorption. Normal-weight individuals were included as control participants. The blood biochemical indicators for detecting bone formation were ALP, PINP, and OCN, while the blood biochemical indicators for bone resorption were CTX-I, DPD, and NTX, excluding bone-specific alkaline phosphatase. The research category included all observational studies, including case-control studies, cross-sectional studies, and cohort studies.

#### 2.2.2 Exclusion criteria

Articles were excluded if they included people who underwent different types of weight loss surgery or had obesity caused by disease; were written in languages other than English; were conference abstracts, systematic reviews, reviews, review articles, letters, case reports, animal studies, and repeated studies; did not provide full-text access; or the article data were incomplete and could not be obtained from the corresponding author via email.

### 2.2 Study selection and data extraction

The literature screening and data extraction were jointly completed by two evaluators (Haiqi Lin and Xiaohua Liu). After deduplication of the data obtained from the database, the title and abstract were read before preliminary screening. If there was a dispute that could not be resolved after repeated verification, a third evaluator (Binglin Chen) was invited to participate and help reach a consensus. Researchers independently extracted the data and, if necessary, invited a third evaluator to participate. If the data were incomplete, the main research author was contacted for additional information. During the literature screening and data extraction process, two evaluators extracted the following information for each included study: author, year, age of the study subjects, outcome indicators, etc. (see **Table 1**).

**Table 1:** Participants characteristics of included studies.

First author, year	Sample size (N)	Age (years)	Sex (% female)	Bone turnover markers	References
Nassar 2007	OB:20 NW:12	OB: 9.13 ± 3.48 NW: 8.83 ± 2.86	46.9%	OCN	(Nassar et al., 2007)
Reinehr 2010	OB:60 NW:19	OB:10.9 ± 0.3 NW:11.6 ± 0.4	50.4%	OCN	(Reinehr and Roth, 2010)
Evans 2015	OB:100 NW:100	25-40 55-75	52%	PINP CTX-I	(Evans et al., 2015)
Gajewska 2015	OB:45 NW:20	OB:7.8 ± 1.7 NW:7.7 ± 2.1	50.8%	CTX-I	(Gajewska et al., 2015)
Matusik 2015	OB:54 NW:75	OB: 13.21 ± 2.8 NW: 13.08 ± 2.4	51.2%	OCN NTX	(Matusik et al., 2015)
Razny 2017	OB:98 NW:34	OB: 46.7 ± 1.2 NW: 48.1 ± 1.9	74.5%	OCN	(Razny et al., 2017)
Viljakainen 2017	OB:55 NW:65	19.5 ± 2.5	52.5%	PINP CTX-I	(Viljakainen et al., 2017)
Carsote 2019	OB:20 OW:19 NW:17	OB:47.40 ± 4.51 OW:48 ± 5.47 NW:47.52 ± 5.03	100%	ALP OCN PINP	(Carsote et al., 2019)
Maïmoun 2020	OB:38 AN:38 NW:38	OB:21.3 ± 2.9 AN:21.0 ± 3.2 NW:21.0 ± 3.2	100%	OCN PINP CTX-I	(Maïmoun et al., 2020)
Yaylali 2021	OB:73 NW:53	OB: 35 ± 6 NW: 32± 8	100%	ALP OCN	(Yaylali et al., 2021)
Yuan 2023	OB:1653 OW:1039 NW:1432	OB:>64(59-69,10) OW: 63(58-68,10) NO: 63(58-68,10)	70.3%	ALP PINP CTX	(Yuan et al., 2023)
Kim 2024	OB:54 NW:49	OB: 10.2 ± 2.4 NW: 9.8 ± 1.7	55.3%	ALP	(Kim et al., 2023)
Wu 2024	OB:200 NW:200	OB:11.9 ± 4.3 NW:11.97 ± 4.5	50%	PINP β-CTX	(Wu et al., 2024)
Sayharman 2025	OB:14 NW:14	OB:21.00 ± 2.42 NW:20.64 ± 1.50	0	OCN	(Sayharman et al., 2025)

OB, Obesity; OW, Overweight; NW, Normal-weight; ALP, Alkaline phosphatase; PINP, Procollagen type I N- propeptide; OCN, osteocalcin; CTX-I, C-telopeptide cross-linked type I collagen; NTX, N-terminal telopeptide of type 1 collagen.

### 2.3 Risk of bias assessment

Haiqi Lin and Xiaohua Liu independently evaluated the quality of each study. We used the Newcastle Ottawa Scale (NOS) to evaluate case-control and cohort studies and the Joanna Briggs Institute tool (JBI) to evaluate cross-sectional studies (Stang, 2010; Cook and Reed, 2015). The score range for the NOS tool is 0 to 9. A score of 7 or above is designated as a low bias risk, a score of 4–6 is designated as a moderate bias risk, and a score below 4 is designated as a high bias risk. The NOS mainly consists of three modules: selection of study groups, comparability, and exposure/outcome. For cross-sectional studies, we applied a modified scoring approach based on the JBI critical appraisal checklist. Each item was scored according to the degree of compliance: 0 indicated non-compliance, 1 indicated that the item was mentioned but not described in detail, and 2 indicated a detailed and comprehensive description. The total score ranged from 0 to 20. Generally, a score greater than 70% of the total score indicates a low risk of bias. All assessments were independently performed by two investigators. If consensus could not be reached after repeated discussions, a third investigator was invited to resolve the disagreement. Details are provided in **Supplementary File 2**.

### 2.4 Data synthesis

The meta-analysis was conducted using Review Manager 5.4 software. The data included in the study were all continuous, and we extracted the mean and standard deviation (SD) to calculate the 95% confidence intervals (CIs) for the meta-analysis. If there was no standard deviation, the data were converted and calculated as much as possible based on p values or standard errors. According to the Q and I^2^ tests, the heterogeneity between different studies was determined. If P≥0.1 and I^2^<50%, a fixed-effects model was used; otherwise, a random-effects model was used. In the overall study, Review Manager 5.4 software was used to exclude the included studies one by one for sensitivity analysis to verify whether any individual study caused differences in the pooled observations.

## 3 Results

### 3.1 Search flow and study overview

**Figure 1:**
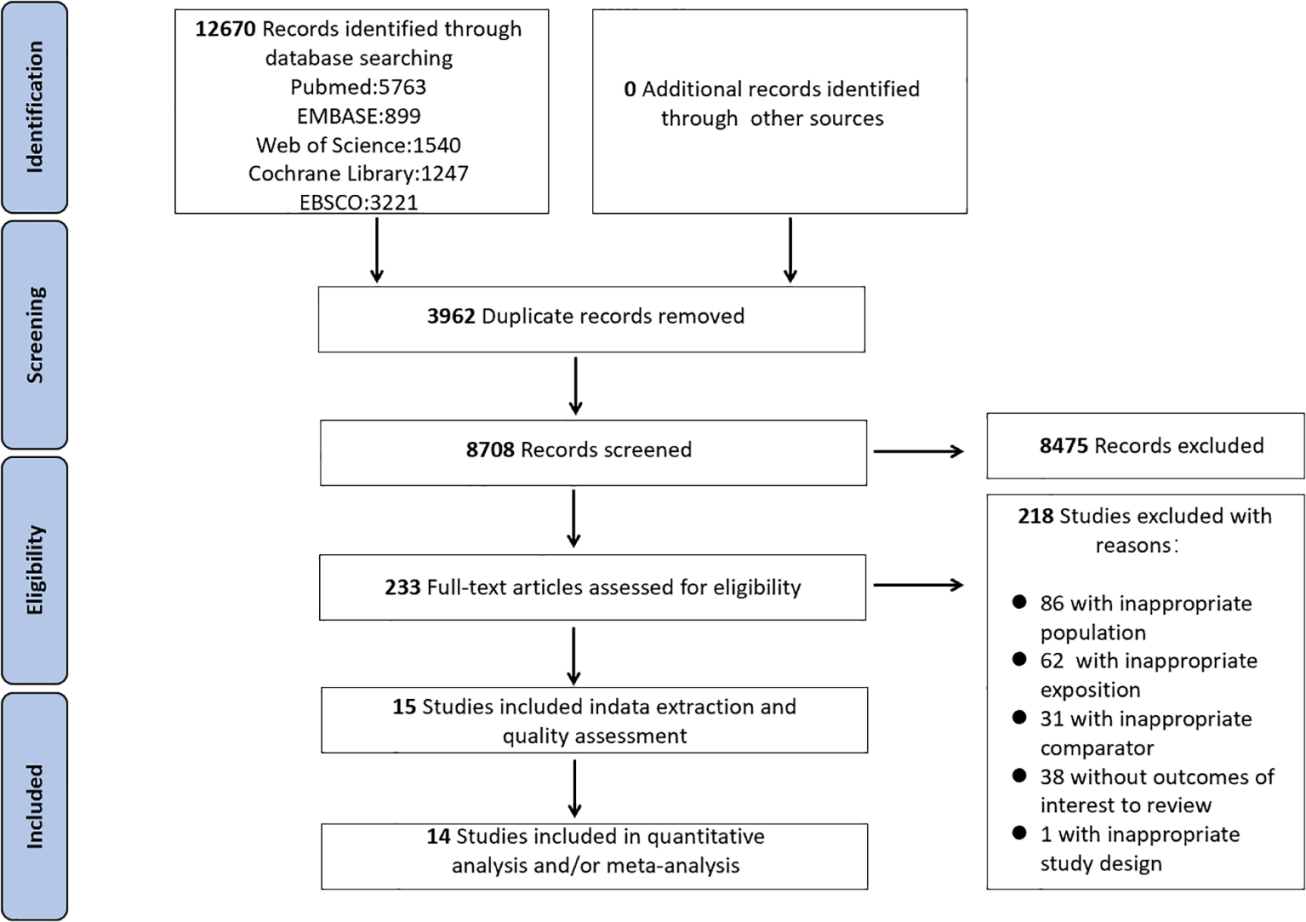
Flow chart of study selection process.

By searching various databases, a total of 12670 articles were identified. After using the management software EndNote 20 to remove duplicate articles, a total of 8708 articles were included. After preliminary screening of the titles and abstracts of the articles, 8475 unrelated articles were excluded, leaving 233 articles. After further reading the entire article, 218 articles were excluded, leaving 15 articles. For one article, we were unable to contact the author to obtain relevant data; therefore, a total of 14 articles were ultimately included in the meta-analysis and data extraction and analysis (see **Figure 1**).

### 3.2 Basic characteristics and quality evaluation of the included studies

The basic characteristics and quality evaluation results of the 14 articles included in themeta-analysis of this study are shown in **Supplementary File 2**, including a total of 4612 subjects, including 2484 individuals with obesity and 2128 normal-weight individuals. Three articles included only women, one article included only men, while ten articles included both men and women. Seven papers were from Europe, five from Asia, one from North America, and one from Africa. Most of the research participants were adolescent, with a small number of middle-aged people and no elderly people. Case-control and cohort studies were evaluated using the NOS, with 2 articles scoring 6 and 2 article scoring 7; the JBI tool was used to evaluate cross-sectional studies, with 2 articles scoring 16, 2 articles scoring 14, 4 articles scoring 14, and 2 articles scoring 13. There was no low-quality literature, so the quality of the literature included in this study was relatively high.

### 3.3 Impact on blood indicators of bone formation

#### 3.3.1 ALP

**Figure 2:**
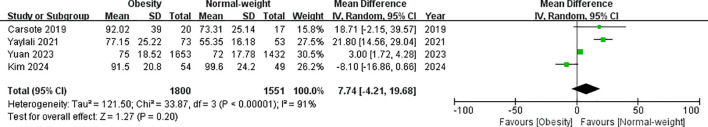
Forest plot showing the difference in ALP levels between the obesity and normal-weight groups in all studies. The black horizontal lines represent the 95% confidence intervals, while the squares represent the point estimates. The black diamonds represent the overall point estimates and 95% confidence intervals.

Four articles (Carsote et al., 2019; Yaylali et al., 2021; Kim et al., 2023; Yuan et al., 2023) reported the impact of obesity on ALP in 3351 patients, including 1800 patients in the obesity group and 1551 patients in the normal-weight group. The results showed heterogeneity (I^2^ = 91, P<0.0001), and a random-effects model was selected for analysis. The results showed no significant differences between the two groups (MD = 7.74, 95% CI -4.21~19.68, P = 0.20; see **Figure 2**). We conducted sensitivity analysis by sequentially removing each individual study to assess the robustness of the pooled effect. The results showed that removing the two study (Yaylali 2021) (Yaylali et al., 2021) had the greatest impact on heterogeneity, reducing the I² value from 91% to 76%. However, even after excluding this study, the pooled effect remained statistically non-significant (MD = 1.53, 95% CI: -8.58 to 11.63, P = 0.77; see **Supplementary Figure 1**). The sequential removal of the remaining studies did not substantially reduce heterogeneity or alter the conclusion of non-significance.

#### 3.3.2 PINP

**Figure 3:**
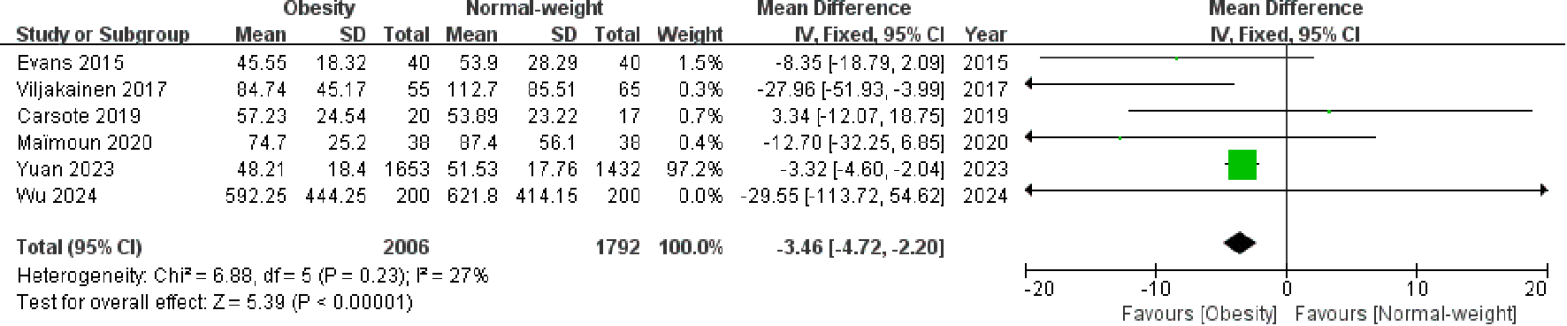
Forest plot showing the difference in PINP levels between the obesity and normal-weight groups in all studies.

Six articles (Evans et al., 2015; Viljakainen et al., 2017; Carsote et al., 2019; Maïmoun et al., 2020; Yuan et al., 2023; Wu et al., 2024) reported the impact of obesity on PINP in 3738 patients, including 2006 patients in the obesity group and 1792 patients in the normal-weight group. The pooled analysis showed low heterogeneity among the included studies, with I^2^ = 27%<50% and P = 0.23 in the Q test; therefore, a fixed-effect model was used. The results revealed that PINP levels were significantly lower in the obesity group than in the normal-weight group (MD= -3.46, 95% CI -4.72~-2.20, P<0.00001; see **Figure 3**).

#### 3.3.3 OCN

**Figure 4:**
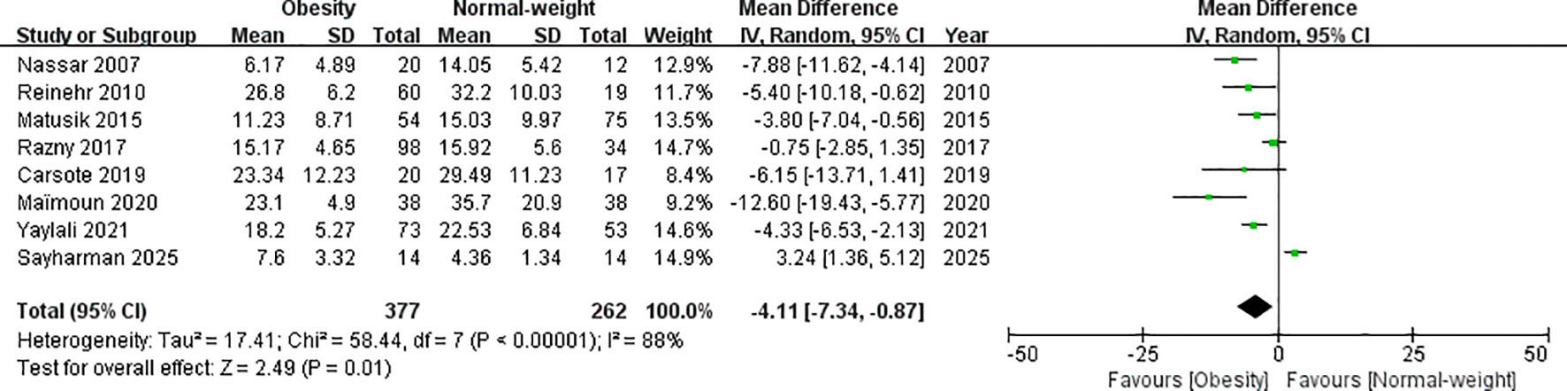
Forest plot showing the difference in OCN levels between the obesity and normal-weight groups in all studies.

Eight articles (Nassar et al., 2007; Reinehr and Roth, 2010; Matusik et al., 2015; Razny et al., 2017; Carsote et al., 2019; Maïmoun et al., 2020; Yaylali et al., 2021; Sayharman et al., 2025) reported the impact of obesity on OCN in 639 patients, including 377 patients in the obesity group and 262 patients in the normal-weight group. The results showed heterogeneity (I^2^ = 88, P<0.0001), and a random-effects model was selected for analysis. The results showed significant differences between the two groups (MD=-4.11, 95% CI -7.34~-0.87, P = 0.01; see **Figure 4**). To explore the sources of heterogeneity, a sensitivity analysis was conducted on the included studies, and the included studies were excluded one by one to evaluate the impact of each study on the OCN effect size. Sensitivity analysis of the included studies revealed no changes in heterogeneity or statistical significance, indicating good stability and reliability of the present study.

To explore the sources of the high heterogeneity in OCN, we conducted a subgroup analysis based on age (using 18 years as the threshold). In the child and adolescent subgroup (≤18 years), the OCN level was significantly lower in the obesity group (MD = -5.57, 95% CI: -8.10 to -3.05, P < 0.0001, see **Supplementary Figure 2**), with very low heterogeneity (I² = 24%). In contrast, in the subgroup with age > 18 years, the pooled effect from five studies did not show statistical significance (MD = -3.18, 95% CI: -7.39 to 1.03, P = 0.14, see **Supplementary Figure 2**), and substantial heterogeneity was observed within this subgroup (I² = 90%). These results suggest that the effect of obesity on serum OCN levels may be age-dependent.

### 3.4 Impact on bone resorption and blood indicators

#### 3.4.1 CTX-I

**Figure 5:**
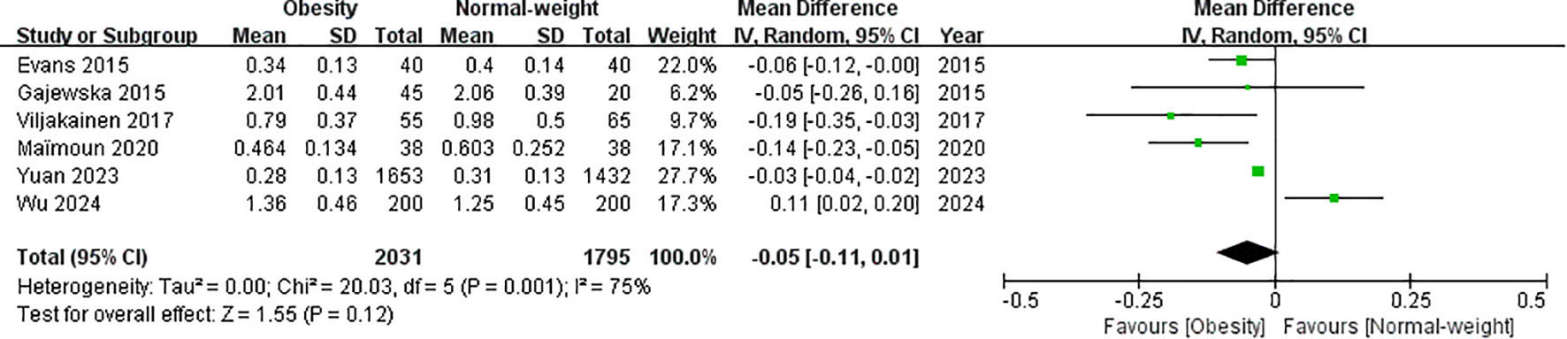
Forest plot showing the difference in CTX-I levels between the obesity and normal-weight groups in all studies.

Six articles (Evans et al., 2015; Gajewska et al., 2015; Viljakainen et al., 2017; Maïmoun et al., 2020; Yuan et al., 2023; Wu et al., 2024) reported the impact of obesity on CTX-I in 3826 patients, including 2031 patients in the obesity group and 1795 patients in the normal-weight group. The pooled analysis showed substantial heterogeneity among the included studies (I² = 75%, Q-test P = 0.001); therefore, a random-effects model was used. The results showed no significant differences between the two groups (MD= -0.05, 95% CI -0.11~0.01, P = 0.12; see **Figure 5**).

#### 3.4.2 NTX

One article (Matusik et al., 2015) reported the impact of obesity on NTX in 129 patients, including 54 patients in the obesity group, and an NTX level of 50.46 ± 60.86. The control group consisted of 75 patients, with an NTX content of 49.21 ± 44.88. The results showed that the obesity group exhibited higher levels of NTX, but the difference was not statistically significant.

## 4 Discussion

In the regulation of bone homeostasis, osteoblasts are responsible for extracellular matrix synthesis, osteoclasts continuously remove old bone, while osteocytes play a key role in modulating skeletal responses to hormonal and mechanical stimuli. As a chronic disease with a high incidence rate and mortality, obesity has attracted extensive attention from researchers (Kolotkin et al., 2001). In recent years, the incidence of obesity and osteoporosis has been continuously increasing, and their interactions with bone metabolism are complex and not fully understood (Conway and Rene, 2004; Gkastaris et al., 2020). There are reports that obesity is a protective factor for bone mass, while some researchers have come to the opposite conclusion: obesity causes an imbalance in the activity of osteoblasts and osteoclasts, leading to bone loss and serious damage to bone health (Proietto, 2020; Forte et al., 2023). The effects of obesity on bone homeostasis and bone cell function have not been studied in detail (Cao, 2011). Compared with a single study, integrating all relevant studies can more accurately evaluate the impact of obesity on bone metabolism indicators. Therefore, this study used a meta-analysis method to evaluate changes in blood biochemical indicators of bone status in individuals with obesity. Due to the influence of circulating estrogen levels on postmenopausal women, which can lead to bone loss, studies evaluating this population were not included in this analysis (Ribot et al., 1987; Papakitsou et al., 2004; Ostrowska et al., 2014; López-Gómez et al., 2022).

Obesity is characterized by the superimposition of mechanical and biochemical effects. Whether it has adverse effects on bone health has been a controversial topic. Studies have shown that biochemical markers of bone turnover are affected by obesity. There are few studies on the relationships between obesity and the bone markers ALP, PINP, OCN, CTX-I, DPD, and NTX, and the results are inconsistent (Geserick et al., 2020). This meta-analysis summarized the data of 14 studies and evaluated the relationship between obesity and bone-turnover biochemical indicators in 4612 subjects, including 2484 individuals with obesity and 2128 normal-weight individuals. Three articles included only women, one article included only men, while ten articles included both men and women. Most of the study participants were adolescent, with a small number of middle-aged people, excluding elderly people. Studies have shown that the evaluation of biochemical markers of bone turnover is very important for understanding normal biological processes and evaluating the treatment of osteoporosis (Choi et al., 2021).

ALP is primarily secreted by hepatocytes, bile duct epithelial cells and osteoblasts. In healthy adults, bone-derived ALP constitutes approximately half of the total ALP activity in serum. Under certain physiological or pathological conditions, the level of liver-derived ALP may be mildly elevated (Whitaker et al., 1977; Czerwińska et al., 2007). When the activity of osteoblasts increases, osteoblasts secrete and release ALP, which is involved in bone calcification and is partially released into the bloodstream. Therefore, ALP levels are considered to be positively correlated with osteogenic activity (Hekkelman, 1967). High expression of ALP is an early marker of osteoblast differentiation and maturation. When ALP activity is enhanced, bone formation is intensified, promoting the mineralization of the bone matrix (Thies et al., 1992; Rickard et al., 1996; Bi et al., 2013). The study showed no significant difference in ALP levels between the control and obesity groups. The observed heterogeneity in ALP results may be attributed to the broad age range of the included studies, which encompassed children, adults, and middle−aged or older adults, as different life stages are associated with distinct physiological levels of ALP. Additionally, disparities in gender distribution and substantial variations in sample size across studies may have led to larger−sample studies disproportionately influencing the pooled effect estimate.

PINP is produced by the decomposition of the amino terminus of procollagen, accounting for more than 90% of bone organic components, reflecting the synthesis rate of collagen. It can be stably present in the blood and is not affected by diet or hormone levels in the body. It is a good marker for bone formation (Eastell and Hannon, 2008; Vasikaran et al., 2023). Serum PINP is influenced mainly by changes in bone turnover and is the best marker for reflecting bone turnover. It is widely used in clinical practice to predict bone loss (Ueda et al., 2002; Koivula et al., 2012; Masik et al., 2021). The results showed that individuals with obesity exhibited significantly lower levels of PINP (P<0.05), indicating a significant decrease in PINP content and osteogenic activity in the body during obesity. This finding indicates that the association between reduced serum PINP levels in obesity demonstrates a consistent trend across studies, suggesting that bone formation activity may be suppressed in the context of obesity.

OCN is specifically produced by osteoblasts and is the most abundant noncollagen protein in bones, consisting of only 49 amino acids (46 in mice) (García-Martín et al., 2013; Komori, 2020; Wang et al., 2021). Most OCN is embedded in the bone matrix, while some is secreted into the blood. Its concentration can be measured in serum, and its level can reflect human bone formation to a certain extent. OCN has been used as a marker of early-stage osteogenic differentiation (Vaes et al., 2010; Zhang et al., 2015; Wang et al., 2022). Research has shown that the metabolic activity of bones is also closely linked to energy-related organs such as fat and pancreatic islets through circulating OCN. Through the whole-body release of OCN, bones transmit their energy needs to other organs to meet their energetic necessities (Chen et al., 2012; Wei and Karsenty, 2015). Although OCN is a sensitive indicator of bone formation, its clinical interpretation remains complex and may be influenced by age, growth status, hormonal regulation, and metabolic conditions. The results of this study showed that individuals with obesity exhibited significantly lower OCN levels (P<0.05), indicating a decrease in osteogenic activity. There was significant heterogeneity in this result. By excluding the included studies one by one, it was found that there were still significant differences between the two groups, indicating that the pooled results were relatively stable. To investigate the sources of high heterogeneity in OCN, we conducted a subgroup analysis based on age. The results revealed that the effect of obesity on OCN is age-dependent. OCN levels were significantly reduced in children and adolescents with obesity, whereas no significant difference was observed in the adult subgroup. This finding suggests that the obesity-associated reduction in OCN may be more pronounced during growth and development, a period characterized by active bone turnover and greater susceptibility to metabolic disturbances. In adults, however, hormonal status, fat distribution, insulin resistance, inflammatory status, and other metabolic factors may influence the relationship between obesity and OCN. Therefore, although obesity appears to be associated with lower overall OCN levels, this conclusion should be interpreted in an age-dependent manner.

Bone turnover markers may display dynamic responses to clinical changes in a given disease state. CTX-I is an important biomarker for bone resorption and is recommended for clinical use by the International Osteoporosis Foundation and the International Federation of Clinical Chemistry. It reflects the bone resorption activity of osteoclasts, and an increase in CTX-I content in serum or urine usually indicates an increase in the bone matrix degradation rate, i.e., increased bone resorption, commonly observed in diseases such as osteoporosis (Cremers and Farooki, 2011; Szulc, 2018). The results of this study indicate that serum CTX-I levels in the obesity group were significantly lower than those in the normal-weight group, although substantial heterogeneity was observed. This finding contrasts with the traditional view that obesity may increase bone resorption through chronic inflammation. The effect of obesity on CTX-I may be influenced by multiple factors, including age, gender, and assay methodology. Future studies should aim to clarify the impact of obesity on bone resorption markers and integrate other bone turnover biomarkers as well as imaging indicators to provide a more comprehensive understanding of the relationship between obesity and bone metabolism.

The cross-linked N-terminal peptide of NTX is a metabolite of type I collagen, which is the main component of the bone matrix (Lung et al., 2002; Hamano, 2006; Lipton et al., 2011). NTX is a low molecular weight peptide containing urinary pyridinoline (Pyr) and urinary deoxypyridinoline (D-Pyr) and is a stable final product that appears in urine after bone degradation. Multiple studies have confirmed a significant negative correlation between urinary NTX/Cr and bone mineral density (BMD), which is a specific and sensitive indicator of bone resorption. Clinically, there is an increase in NTX levels in patients with osteoporosis, primary hyperparathyroidism, osteoarthritis, hyperthyroidism, tumor bone metastasis, and multiple myeloma (Delmas, 1999; Lund et al., 2010; Jablonka et al., 2014). This study included only one observational study on the correlation between obesity and NTX concentration, which revealed that the obesity group exhibited greater NTX levels, but the difference was not significant (P>0.05). The author compared male and female individuals with obesity and normal-weight controls separately and found that girls with obesity exhibited higher levels of NTX, but there was no significant difference in boys. At the same time, the author noted that the bone turnover rate (OCN/NTx) was only significantly reduced in girls with obesity, indicating that sex differences may have a significant impact on bone resorption indicators (Matusik et al., 2015).

DPD is a byproduct of the cross-linking of single collagen peptides after type I collagen decomposition, is released by osteoclasts and is almost exclusively present in bone and dentin, with high bone specificity (Delmas, 1993; Zhan et al., 1999). It can be measured in urine, but due to its daily variation pattern, early morning spot urine is the preferred sample collection method. By measuring plasma DPD levels, bone resorption can be evaluated, bone loss rates and the resulting fracture risk can be predicted, and bone mass can be estimated (Garnero and Delmas, 1996; Shidara and Inaba, 2009). El Dorry et al (El-Dorry et al., 2015). showed that DPD cannot be used as an early indicator. In our meta-analysis, no literature suitable for this topic was found, indicating that further in-depth research is needed for the testing of this indicator in individuals with obesity to provide a theoretical basis for subsequent treatment of obesity.

The results of this meta-analysis indicate that bone homeostasis is imbalanced in individuals with obesity. Bone turnover markers play an irreplaceable role in the differential diagnosis, fracture risk assessment, and monitoring and treatment of osteoporosis. Compared with BMD, bone turnover markers can reflect the state of bone turnover in a timely manner. However, according to the current research, bone turnover markers cannot be used to detect osteoporosis and can only be used to differentiate and diagnose secondary osteoporosis. However, this is a promising research direction, and more research is needed to explore the diagnostic value of bone turnover markers. This study aimed to investigate changes in bone turnover markers in individuals with obesity. Our meta-analysis revealed that levels of key bone formation markers (PINP and OCN) were significantly lower in obesity group compared to normal-weight group. While a trend of decrease was also observed in the bone resorption marker CTX-I, its robustness requires further validation due to heterogeneity among studies. No significant differences were found in ALP or NTX levels. These findings suggest a generally suppressed state of bone turnover in obesity. The potential reasons for these inconsistent results are the heterogeneity of the included population (age, sex, etc.), as well as the preanalysis and analysis variability of bone turnover marker measurements (i.e., fasting state and measurement time of the day, serum or plasma measurements, measurement types and specimen storage conditions), and the possibility that bone blood biochemical indicators may be influenced by body composition and nutritional status (Mele et al., 2022). In addition, although obesity is a well-known metabolic disorder and there have been many studies on the possible diseases caused by obesity, the new concept of obesity (i.e., weight itself is not as important as the percentage of body fat) is not well known or studied.

### 4.1 Strengths and limitations

Our systematic review and meta-analysis has several strengths, including an exhaustive search strategy and the assessment of multiple biochemical markers of bone turnover. The methodological quality of all included studies was also assessed using validated tools, including the NOS for case-control and cohort studies and the JBI tool for cross-sectional studies.

Several limitations should be acknowledged. First, the relatively small number of included studies limited our ability to perform detailed subgroup analyses according to sex, age, BMI category, obesity severity, and body fat distribution. Second, important demographic gaps existed, as the included studies mainly involved children, adolescents, and a limited number of adults, whereas data on elderly individuals were limited. In addition, studies involving postmenopausal women with obesity were not included, which limits the generalizability of our findings to this high-risk population. The effect of type 2 diabetes could not be specifically evaluated, although it commonly coexists with obesity and may independently affect bone quality. Finally, differences in fasting status, sample collection time, specimen type, storage conditions, and assay methods may have contributed to heterogeneity. Future studies should adopt standardized protocols for bone turnover marker collection and measurement to reduce preanalytical variability and improve comparability across studies.

## 5 Conclusions

This meta-analysis suggests that obesity is associated with suppressed bone turnover, mainly reflected by reduced levels of bone formation markers. PINP levels were significantly decreased in individuals with obesity, whereas the reduction in OCN appeared to be age-dependent, with significant suppression observed in children and adolescents but not in adults. However, the robustness of the bone resorption results is insufficient and requires further validation through additional studies. The interaction between obesity and bone homeostasis is complex because there are many factors that interfere with it. Increasing amounts of data indicate that obesity is harmful to bone health, despite the potential positive impact of weight gain on the mechanical load of bones. Therefore, the concept that obesity can prevent bone loss is not entirely clear. Moreover, biochemical markers of bone turnover cannot replace dual energy X-ray detection of bone density, which is the gold standard for the diagnosis of osteoporosis. Comprehensive analysis must be conducted based on the patient’s medical history, clinical symptoms, physical examination, and other test results. In the future, more research is needed to evaluate their relationships and propose measures to prevent their negative effects on bones. Moreover, understanding the relationship between obesity and bone metabolism may help identify new molecular targets that can increase osteoblast production while inhibiting adipogenesis and/or reducing osteoclast formation. Further research on the relationship between obesity and bone homeostasis may lead us to develop new treatment measures to prevent osteoporosis caused by obesity. Due to the overall poor methodological quality of its systematic evaluation, more high-quality experiments need to be conducted, and the quality of research methodology and reporting needs to be standardized to provide more high-level evidence-based medical evidence for the clinical application of changes in bone homeostasis in individuals with obesity.
