# Supplementary material for: Obesity is associated with suppressed bone turnover: a systematic review and meta-analysis
Source: Front Physiol. 2026 Jun 10;17:1793838. doi: 10.3389/fphys.2026.1793838 (PMC13290598; doi:10.3389/fphys.2026.1793838)
Supplement: Supplementary file 1 [file SupplementaryFile1.docx]

**Supplementary File 1.** Search strategies used for each database.

**1.PubMed**

#1 Obesity 1048000 results

(((((("Obesity"[MeSH Terms]) OR ("Body Mass Index"[MeSH Terms])) OR ("obesity"[Title/Abstract])) OR ("Body Mass Index"[Title/Abstract])) OR ("fatty"[Title/Abstract])) OR ("excess fat"[Title/Abstract])) OR ("body fat*"[Title/Abstract])

#2 Outcome:bone formation 235969 results

(((((((((("Osteogenesis"[MeSH Terms]) OR (“Alkaline Phosphatase”[MeSH Terms])) OR ("Osteocalcin"[MeSH Terms])) OR (“Osteogenesis”[Title/Abstract])) OR (“Ossification*”[Title/Abstract])) OR ("bone formation"[Title/Abstract])) OR ("bone metabolism"[Title/Abstract])) OR (“bone turnover”[Title/Abstract])) OR ("Alkaline Phosphatase"[Title/Abstract])) OR ("procollagen type I N-terminal peptide"[Title/Abstract])) OR ("Osteocalcin"[Title/Abstract])

#3 Outcome:bone resorption 69206 results

((((("Bone Resorption"[MeSH Terms]) OR ("bone resorption*"[Title/Abstract])) OR ("osteoclastogenesis"[Title/Abstract])) OR ("collagen type I trimeric cross-linked peptide"[Title/Abstract])) OR ("deoxypyridinoline"[Title/Abstract])) OR ("N-terminal telopeptide of type I collagen"[Title/Abstract])

#4 #2 OR #3 282236 results

#5 #1 AND #4 8985 results

#6 humans NOT animals

#7 #5 AND #6 5763 results

**2.EMBASE**

#1 Obesity 1884750 results

'obesity'/exp OR 'body mass index'/exp OR 'obesity':ab,ti OR 'body mass index':ab,ti OR 'fatty':ab,ti OR 'excess fat':ab,ti OR 'body fat*':ab,ti

#2 Outcome:bone formation 406225 results

'osteogenesis'/exp OR 'alkaline phosphatase'/exp OR 'osteocalcin'/exp OR 'bone formation':ab,ti OR 'bone metabolism':ab,ti OR 'bone turnover':ab,ti OR 'alkaline phosphatase':ab,ti OR 'procollagen type i n-terminal peptide':ab,ti OR 'osteocalcin':ab,ti OR 'osteogenesis':ab,ti OR 'ossification*':ab,ti

#3 Outcome:bone resorption 119778 results

'bone resorption'/exp OR 'bone resorption*':ab,ti OR 'osteoclastogenesis':ab,ti OR 'collagen type i trimeric cross-linked peptide':ab,ti OR 'deoxypyridinoline':ab,ti OR 'n-terminal telopeptide of type i collagen':ab,ti

#4 #2 OR #3 483384 results

#5 #1 AND #4 31108 results

#6 humans NOT animals

#7 #5 AND #6 899 results

**3.Web of science**

#1 Obesity 1205347 results

TS=(“obesity” OR "Body Mass Index" OR "fatty" OR "excess fat" OR "body fat*")

#2 Outcome:bone formation 172500 results

TS=(“Osteogenesis” OR “Ossification*” OR "bone formation"OR "bone metabolism"OR

"bone turnover" OR "Alkaline Phosphatase" OR "procollagen type I N-terminal peptide" OR "Osteocalcin")

#3 Outcome:bone resorption 34795 results

TS=("bone resorption*" OR "osteoclastogenesis" OR "collagen type I trimeric cross-linked peptide" OR "deoxypyridinoline"OR "N-terminal telopeptide of type I collagen")

#4 #2 OR #3 190917 results

#5 #1 AND #4 11341 results

#6  TS=(humans NOT animals)

#7 #5 AND #6 1540 results

**4.Cochrane Library**

＃1 Obesity 139650 results

MeSH descriptor: [Obesity] explode all trees OR MeSH descriptor: [Body Mass Index] explode all trees OR (“obesity”):ti,ab,kw OR ("Body Mass Index"):ti,ab,kw OR ("fatty"):ti,ab,kw OR ("excess fat"):ti,ab,kw OR ("body fat*"):ti,ab,kw

＃2 Outcome:bone formation 19803 results

MeSH descriptor: [Osteogenesis] explode all trees OR MeSH descriptor: [Alkaline Phosphatase] explode all trees OR MeSH descriptor: [Osteocalcin] explode all trees OR

("Osteogenesis"):ti,ab,kw OR (“Ossification*”):ti,ab,kw OR ("bone formation"):ti,ab,kw OR ("bone metabolism"):ti,ab,kw OR ("bone turnover"):ti,ab,kw OR ("Alkaline Phosphatase"):ti,ab,kw OR ("procollagen type I N-terminal peptide"):ti,ab,kw OR ("Osteocalcin"):ti,ab,kw

＃3 Outcome:bone resorption 6883 results

MeSH descriptor: [Bone Resorption] explode all trees OR ("bone resorption*"):ti,ab,kw OR ("osteoclastogenesis"):ti,ab,kw OR ("collagen type I trimeric cross-linked peptide"):ti,ab,kw OR ("deoxypyridinoline"):ti,ab,kw OR ("N-terminal telopeptide of type I collagen"):ti,ab,kw

#4 #2 OR #3 23203results

#5 #1 AND #4 2635 results

#6 humans NOT animals

#7 #5 AND #6 1247 results

**5. EBSCO**

＃1 Obesity 3791595 results

SU obesity OR SU Body Mass Index OR TI Body Mass Index OR AB Body Mass Index OR AB fatty OR TI fatty OR AB excess fat OR TI excess fat OR AB body fat* OR TI body fat* OR AB obesity OR TI Obesity

＃2 Outcome:bone formation 832402 results

SU Osteogenesis OR SU Alkaline Phosphatase OR SU Osteocalcin OR TI Osteogenesis OR AB Osteogenesis OR AB Ossification* OR TI Ossification* OR AB bone formation OR TI bone formation OR AB bone metabolism OR TI bone metabolism OR AB bone turnover OR TI bone turnover OR AB Alkaline Phosphatase OR TI Alkaline Phosphatase OR AB procollagen type I N-terminal peptide OR TI procollagen type I N-terminal peptide OR AB Osteocalcin OR TI Osteocalcin

＃3 Outcome:bone resorption 190482 results

SU Bone Resorption OR AB bone resorption* OR TI bone resorption* OR AB osteoclastogenesis OR TI osteoclastogenesis OR AB collagen type I trimeric cross-linked peptide OR TI collagen type I trimeric cross-linked peptide OR AB deoxypyridinoline OR TI deoxypyridinoline OR AB N-terminal telopeptide of type I collagen OR TI N-terminal telopeptide of type I collagen

#4 #2 OR #3 936819 results

#5 #1 AND #4 32530 results

#6 humans NOT animals

#7 #5 AND #6 3221 results
