# Supplementary material for: Obesity is associated with suppressed bone turnover: a systematic review and meta-analysis
Source: Front Physiol. 2026 Jun 10;17:1793838. doi: 10.3389/fphys.2026.1793838 (PMC13290598; doi:10.3389/fphys.2026.1793838)
Supplement: Supplementary file 2 [file SupplementaryFile2.docx]

**Supplementary File 2.** Characteristics of the population included in the study, biochemical indicators of bone turnover, and quality scores.

| **References** | **Country** | | **Study design** | **Obesity criterion** | | | **Number of patients(N)** | **Fasting** | **Specimen type** | **Assay used** | **Quality scorea** |
| --- | --- | --- | --- | --- | --- | --- | --- | --- | --- | --- | --- |
| Nassar 2007 | Egypt | | Case-control study (32) | OB:>p95th percentile  NW: BMI within normal percentiles | | | 32 | Not mentioned | OCN: Serum | EASIA | 7 |
| Reinehr 2010 | Germany | | CSS (79) | | OB: obesity according to IOTF criteria; NW: normal weight | | 79 | Yes | OCN: Serum | Chemiluminescent immunometry | 14 |
| Evans 2015 | UK | | Cross sectional case-control study (200) | | OB: BMI≥30  NW:18.5-24.9 | | 200 | Yes | PINP: Serum  CTX-I: Serum | Automated electrochemiluminescence immunoassay, Cobas e411, Roche Diagnostics | 6 |
| Gajewska 2015 | | Poland | CSS (65) | | OB: BMI z-score> 2  NW: BMI z-score between -1 and +1 | | 65 | Yes | CTX-I: Serum | ELISA | 16 |
| Matusik 2015 | | Poland | CSS (129) | | | OB: BMI≥26.5  NW: BMI<26.5 | 129 | Yes | OCN: Serum  NTX: Serum | OCN: Quantitative  sandwich enzyme immunoassay technique  NTX: ELISA | 16 |
| Razny 2017 | | Poland | CSS (132) | | | OB: 30<BMI<40  NW: <30 | 132 | Yes | OCN: Serum | EASIA | 14 |
| Viljakainen 2017 | | Finland | CSS (120) | | | OB: median 40 kg/m2  NW: non-obese controls | 120 | Yes | PINP, CTX-I: Serum | IDS-iSYS automated analyzer | 15 |
| Carsote 2019 | | Romania | CSS (56) | | | OB: BMI:30-34.9  OW: 25-29.9  NW: 18.5-24.9 | 37 | Not mentioned | ALP, OCN, PINP: Serum | ALP: colorimetric assay; CrossLaps: electrochemiluminescence assay  OCN: electro-  chemiluminescence method  P1NP: ECLIA | 13 |
| Maïmoun 2020 | | France | Case control study (114) | | | OB: BMI>30  NW:18<BMI<25  AN: BMI<17.5 | 76 | Yes | OCN, PINP, CTX-I: Serum | Cobas 6000 | 6 |
| Yaylali 2021 | | Turkey | CSS (126) | | | OB: BMI >30  NW: normal weight controls | 126 | Yes | ALP, OCN: Serum | ALP: Not mentioned OCN: chemiluminescence immunoassay. | 15 |
| Yuan 2023 | | China | CSS (4124) | | | OB: BMI>25.0  OW:22.9-24.9  NW: 18.5-22.9 | 3085 | Not mentioned | ALP, PINP, CTX: Serum | Roche automatic analyzer | 14 |
| Kim 2024 | | Korea | CSS (103) | | | OB: ≥95th  NW:15th<BMI<84th | 103 | Yes | ALP: Serum | Not mentioned | 14 |
| Wu 2024 | | China | Case-control study  (400) | | | OB: ≥p95  NW: p5 <BMI<p95 | 400 | Yes | PINP, β-CTX: Serum | electrochemiluminescence, Roche Cobas8000 E602 | 7 |
| Sayharman  2025 | | Turkey | CSS (28) | | | OB: 30.0<BMI<34.9  NW: BMI 18.5<BMI<24.9 | 28 | Yes | OCN: Plasma | ELISA | 13 |

CSS: Cross-sectional study; CS: Cohort study. OB: Obesity; OW: Overweight; NW: Normal-weight; BMI: Body Mass Index; ALP: Alkaline phosphatase; PINP: Procollagen type I N- propeptide; OCN: osteocalcin; CTX-I: C-telopeptide cross-linked type I collagen; NTX: N-terminal telopeptide of type 1 collagen; BMI is expressed in kg/m^2^.

We used Newcastle Ottawa Scale (NOS) to evaluate case-control and cohort studies, and Joanna Briggs Institute tool (JBI) to evaluate cross-sectional studies.
