# Supplementary material for: Obesity is associated with suppressed bone turnover: a systematic review and meta-analysis
Source: Front Physiol. 2026 Jun 10;17:1793838. doi: 10.3389/fphys.2026.1793838 (PMC13290598; doi:10.3389/fphys.2026.1793838)
Supplement: Supplementary file 3 [file SupplementaryFile3.docx]

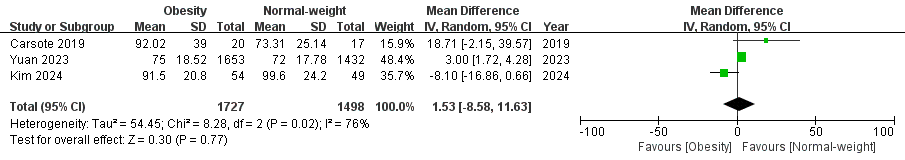


Supplementary figure 1 Forest plot of the sensitivity analysis comparing ALP levels between obesity and normal-weight groups


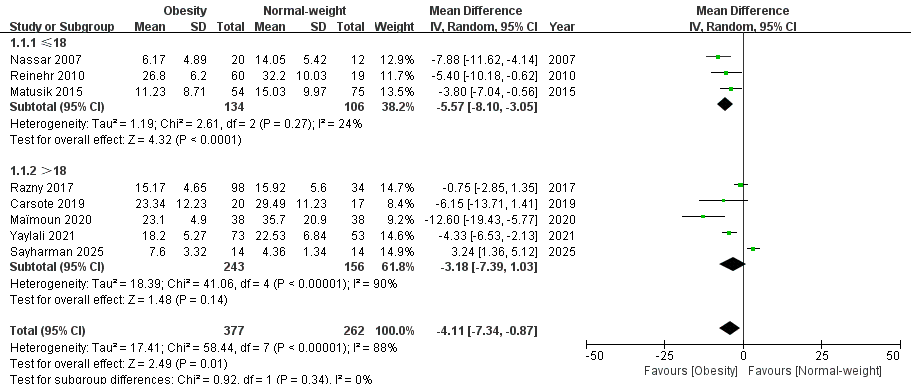


Supplementary figure 2 Forest plot showing subgroup analysis by age of the difference in OCN levels between obesity and normal-weight groups
